# Observational study of Interleukin-21 (IL-21) does not distinguish Kawasaki disease from other causes of fever in children

**DOI:** 10.1186/s12969-017-0163-3

**Published:** 2017-04-20

**Authors:** Rachel Engelberg, Meghan Martin, Brian H. Wrotniak, Mark Daniel Hicar

**Affiliations:** 1 Jacobs School of Medicine and Biomedical Sciences, University at Buffalo, Buffalo, NY USA; 2 Department of Pediatrics, University at Buffalo, Buffalo, NY USA

**Keywords:** Kawasaki Disease, Biomarker, Interleukin-21, IL-21, Pediatric Vasculitis

## Abstract

**Background:**

Kawasaki disease (KD) is a febrile childhood vasculitis of unknown etiology. The diagnosis is highly concerning as over a quarter of children who fail to receive timely treatment with intravenous immunoglobulin (IVIG) will develop coronary aneurysms. Diagnosis relies on proper symptomatology and is supported by non-specific markers of inflammation. Previous studies have identified elevated plasma levels of interleukin-21 (IL-21) as a sensitive and specific biomarker in KD. The aim of this study is to assess the validity of IL-21 as a diagnostic biomarker for KD in febrile children in North America.

**Methods:**

Plasma samples were collected from children who presented to an urban Emergency Department in North America. IL-21 levels were measured using commercial ELISA kits in 12 KD versus 60 controls subjects.

**Results:**

Our study shows that IL-21 levels were non-specifically elevated across all febrile children, irrespective of KD diagnosis. Length of fever prior to sample collection does not correlate with IL-21 levels. Other inflammatory markers and laboratory values were also compared to IL-21 and show no significant correlation.

**Conclusions:**

Since IL-21 is elevated non-specifically in this cohort, our data supports that IL-21 is not an appropriate biomarker for diagnosis of KD in North American pediatric populations.

**Electronic supplementary material:**

The online version of this article (doi:10.1186/s12969-017-0163-3) contains supplementary material, which is available to authorized users.

## Background

Kawasaki disease (KD) is the leading cause of acquired heart disease in children [1, 2]. Nearly one quarter of untreated KD children will go on to develop coronary aneurysms. The classic KD presentation is a systemic vasculitis with minimum of 5 days of fever and 4 of the 5 following clinical criteria; conjunctivitis, rash, distal extremity swelling, oral mucous membrane inflammation, and non-generalized lymphadenopathy [3]. Both incomplete forms, presenting with less than 4 criteria, and atypical variants, presenting classically but with an additional clinical finding not typically seen with KD, have a similar coronary arteritis propensity to classic KD presentations [4]. Treatment with intravenous immunoglobulin (IVIG) given prior to the 10^th^ day of illness helps to prevent the development of coronary aneurysms [5].

Several studies have supported a role for B cell responses in the pathogenesis of KD. Trans-placental passage of maternal antibodies is thought to be protective and explain the paucity of cases in infancy [6]. Recent studies support a protective role of breastfeeding [7]. Genome-wide genetic marker association studies show that specific polymorphisms in CD40 and in the B lymphoid tyrosine kinase genes associate with KD [8, 9]. IgA+ plasma cells are shown within inflammatory infiltrates in the vessel walls of aneurysms from KD children [10, 11], and depleted in the peripheral blood compartment [12]. These IgA+ cell rich aneurysmal infiltrates are proposed to be specific responses to an infiltrating pathogen.

IL-21, produced mainly by T cells and Natural Killer cells [13, 14], has recently been shown to be a specific marker in KD in a Korean cohort of children [15]. In this study, IL-21 was elevated compared to a control group of children with prolonged fevers thought to be due to mononucleosis. This finding further supports a role of B cell activation in KD, since IL-21 modulates immunoglobulin isotype switching and is involved in the differentiation of both naïve and memory B cells into mature plasma cells [16]. Only one other published study, focusing on the chemokine IP-10 (CXCL10), has included IL-21 levels in there published data [17]. Unfortunately, the cytokine bead array used was comparably much less sensitive than the published ELISA-based data [15], making direct comparison difficult.

We wished to assess if IL-21 could be used as a marker of KD in a North American cohort of febrile children to distinguish KD from other febrile presentations. We recruited 12 KD and 60 control subjects between 9 months and six years of age through an urban Emergency Department. Within this manuscript we detail our results that show that IL-21 levels were non-specifically elevated across all febrile children, irrespective of KD diagnosis.

## Methods

### Enrollment

This is a single site observational (unmatched case–control) study of children with suspected KD compared to other febrile children. Subjects consisted of children aged nine months to six years of age who presented to the Emergency Department of Women and Children’s Hospital of Buffalo (WCHOB) between March 2014 and March 2015. Patients in this age group with a planned blood draw as part of their emergency room evaluation were eligible if they had fever (>100.9 °F or 38.2 °C) at home (within 24 h of seeking care) or confirmed in the emergency room and at least one of the following symptoms: rash, mucous membrane changes, extremity changes, conjunctivitis or a single isolated enlarged lymph node. Patients were also eligible for enrollment if they were sent to the emergency room with the specific concern for Kawasaki disease. After parental informed consent was obtained, during clinical blood collection, an additional 5–10 ml of blood was collected. This study successfully enrolled 72 subjects. University at Buffalo’s Human Research Protection Program institutional review board approval was obtained prior to the initiation of the study and has been modified and renewed on a yearly basis (MODCR00000185).

Notable exclusions to prevent effects of excessive blood draws included prior study enrollment within two months, chronic or active blood borne infection such as HIV, HBV or HCV, chronic anemia, excessive blood loss or previous multiple blood draws within 8 weeks prior to study enrollment. Symptoms occurring at ED presentation, vital signs, and demographic information were recorded on the study data collection form. Access to the medical record was included in the consent to allow for review of individual medical records to assign enrolled subjects as KD or controls. Assignment to the KD group required the following: admittion to the hospital, an infectious disease consultant agreed with the diagnosis of KD, and the subject having received immunoglobulin therapy. Notably, there were no controversies in assignment between the primary team and infectious disease consultant, no control subject received IVIG, and most control subjects were not admitted.

### ELISA tests

During peripheral blood mononuclear cell (PBMC) isolation for a separate study, diluted plasma (roughly 40% with PBS) was withdrawn and stored in a −80 °C freezer. The Human IL-21 ELISA kit (Ready-SET-Go! Kit, Affymetrix, San Diego) with a published sensitivity range of 8–1000 pg/mL was used in this study. The manufacturer’s protocol was followed and is briefly summarized. Anti-Human IL-21 capture antibody was bound to 96-well ELISA plates overnight (o/n) at 4 °C. IL-21 coated plates were washed three times (3×) then blocked (1 h, room temperature (RT)), then washed once. Human IL-21 internal standards (ranging from 15.625 pg/mL to 800 pg/mL) and diluted plasma samples were layered on the plate in duplicate and incubated (o/n, 4 °C). After washing (5×) plates were incubated (1 h, RT) with anti-Human IL-21 Biotin detection antibody. Plates were washed (5×) and incubated (30 min, RT) with Avidin-HRP enzyme. After washing (7×), plates were developed with TMB solution with 2N H_2_SO_4_ acid stop and read at 450 nm in a spectrophotometer.

Plasma was initially diluted with PBS during PBMC isolation (generally roughly 30–40% of full concentrated plasma). Raw ELISA data was converted to final concentrated plasma levels by accounting for this predilution. Measured raw IL-21 levels were divided by plasma dilution (plasma dilution = Volume Blood(1- hematocrit/100)/(Volume blood + Volume PBS added)).

### Data analysis

Descriptive characteristics for study subjects were computed. Most variables were positively skewed, and are, therefore, described using medians, 2.5^th^, and 97.5^th^ percentiles. Categorical variables were reported as proportions in percentage. Separate Mann-Whitney U tests were used to examine differences between patients with KD and controls for clinical variables (see Additional file 1: Table S1). Statistical tests were two-tailed with alpha of 0.05. Analyses were conducted with SYSTAT 13 (SYSTAT Software, 2004).

Enrollment survey and chart review was used to define number of symptoms consistent with KD. Spearman rank correlation coefficients (R) of laboratory value versus IL-21 levels and Mann-Whitney U tests comparing IL-21 levels between comparative groups were analyzed and graphed using Prism software (Graphpad, La Jolla, CA).

## Results

### Levels during fever

Over a period of 1 year, 70 children with fever, including 12 with diagnoses of KD, and two non-febrile subjects with specific concern for KD were enrolled in this study. Median laboratory values showing typical significant differences between KD subjects and controls are as follows: Albumin (2.9 vs 3.8 g/L), CRP (106.8 vs 22.9 mg/L), hematocrit (32.1 vs 34.8%), and hemoglobin (11.0 vs 11.9 g/dL). White blood cell (WBC) count was elevated in KD subjects, but this did not show statistical significance (14.7 vs 10.7 × 10^6^/mL). Platelets were also elevated in KD subjects (386 vs 297 × 10^4^/mL) however, sampling was done relatively early in the course when there is not a typical excessive thrombocytosis [18]. These significant differences showing hypoalbuminema, anemia, and inflammation in the KD subjects is consistent with previous studies on KD [4].

**Fig. 1:**
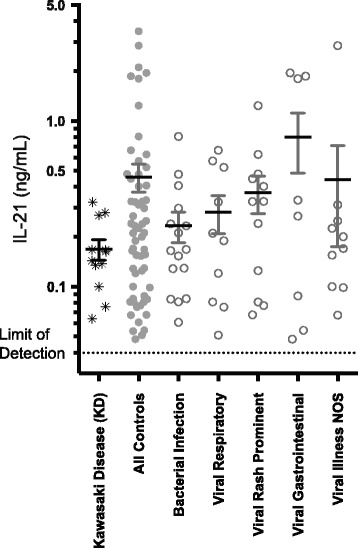
IL-21 levels in KD versus febrile controls. Results represent pooled data of IL-21 of duplicated wells from two separate experiments (see Additional file 1: Table S1 for grouping data). Mean and standard deviations are noted by error bars. IL-21 is graphed on a log^10^ scale to improve display of data points

Comparing IL-21 levels from all control subjects to KD subjects shows control samples had generally higher IL-21 levels which contradicts data supporting IL-21 as a specific marker [15] (Fig. 1). The published sensitivity of the Ready-SET-Go! Kit (Affymetrix, San Diego) was 8.0 pg/mL. All of our samples showed results over the assays limit of detection. This was above 15.6 pg/mL or above 40 pg/mL after adjustment for predilution of the samples. Our background levels were similar to the lower limit of detection of 62.5 pg/mL in previous publication, and our median level in the KD group was also similar to that previously published (499.5 pg/mL) [15]. Repeating analyses of the data with higher level of detection (75 pg/mL), or with values falling below 40 pg/mL as estimated values or set to zero pg/mL resulted in similar results (data not shown).

Notably, only a minority of control samples had elevations of IL-21 outside of the range of KD subjects. To explore if this elevation was being driven by any certain subgroup within the controls, medical records were reviewed and the following clinical sub-groups were defined: bacterial infection, viral respiratory infection, viral rash prominent illness, viral predominant gastrointestinal illness, and not otherwise specified viral illness. The specific diagnoses that make up each of these groups were assigned by chart review and ICD9 discharge diagnosis and are included in Table 1. All control subsets had higher mean values of IL-21 than the KD group and showed significant variability. Compared to KD levels, significant elevation of IL-21 was seen in the category of suspected viral illness (*p* = 0.0074). This data suggests that IL-21 is not specifically elevated in KD, nor is IL-21 elevation specific to a single diagnostic group.

**Table 1:** Clinical diagnoses of control subjects

*Bacterial Infection (16)* ^a^	*Viral Rash Prominent (12)*
Cervical Adenitis (5)	Viral Syndrome (7)
Abscess (2)	Acute Hepatitis
Cellulitis (2)	Irritant Diaper Dermatitis
Pyelonephritis (2)	Eczema Herpeticum
Otitis Media (2)	Viral conjunctivitis
Bacteremia (2)	Hand Food and Mouth Disease
Septic Arthritis	
	*Viral Gastrointestinal (8)*
*Rheumatologic/Vascular (2)*	Viral Gastroenteritis (4)
Henoch-Schönlein Purpura	Diarrhea (3)
Juvenile Idiopathic Arthritis	Parainfluenza
*Viral Respiratory (10)*	*Viral (Not Otherwise Specified) (10)*
Upper Respiratory Infection (4)	Viral Syndrome (8)
Viral Pneumonia (2)	Viral Meningitis
Acute Bronchiolitis	Febrile Neutropenia
Adenovirus	
Influenza A	*Afebrile (2)*
Febrile Seizure	

^a^Numbers in each category are listed in parentheses (). Broad control categories are italicized, with more specific diagnoses underneath each header. Lack of parenthesis indicates that category was only a single event

### Time of fever

**Fig. 2:**
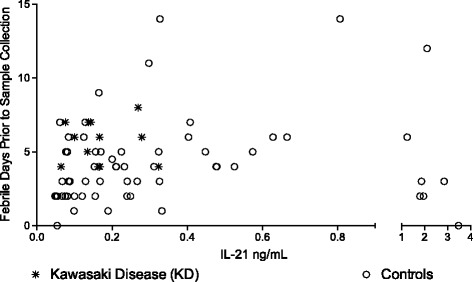
IL-21 in KD and controls in relation to day of fever. Length of fever was calculated by review of the medical record for each subject. IL-21 levels did not have significant correspondence with duration of fever in febrile pediatric patients presenting to the Emergency Department

The previous study collected samples in the acute phase of KD, but did not specifically note length of fever at the point of blood draw [15]. No study to date has analyzed the fluctuations of IL-21 in KD across the acute, subacute, and convalescent stages of disease. However, a number of laboratory parameters have been studied and vary over time during the course of KD [18, 19]. Levels of numerous cytokines, including IL-21, may be influenced by length of illness in other infections. Both Epstein-Barr Virus [20] and Dengue virus infections [21] have shown delays in IL-21 levels that rise past seven days into the infection. KD presentation and response to treatment is also temporally related, as the efficacy of treatment is known to be more predominant in the acute phase of the disease [4]. Since this prolonged presentation may influence serum cytokine levels, and may explain disparity in the published studies, we chose to explore whether length of fever correlated with IL-21 levels. Hospital records were reviewed to estimate length of fever. Length of fever (Fig. 2) was not significantly different between the groups, however the controls have a much wider range of length of fever prior to being enrolled in this study. Notably, one of the highest levels of plasma IL-21, 3.47 ng/mL was seen in one of two enrollees without fever. Calculation of Spearman R coefficients did not reveal any significant correlation to time of fever in either KD or control group.

### Correlations with clinical indices

With incomplete cases of KD (those lacking 4 or 5 major criteria), diagnosis often relies on other clinical laboratory criteria [4]. Supplemental markers of KD used in diagnosis of incomplete KD include low albumin, low hemoglobin and elevations in the following: C-reactive protein (CRP), erythrocyte sedimentation rate (ESR), platelets, white blood cell (WBC) count, and alanine transaminase (ALT) [4].

**Fig. 3:**
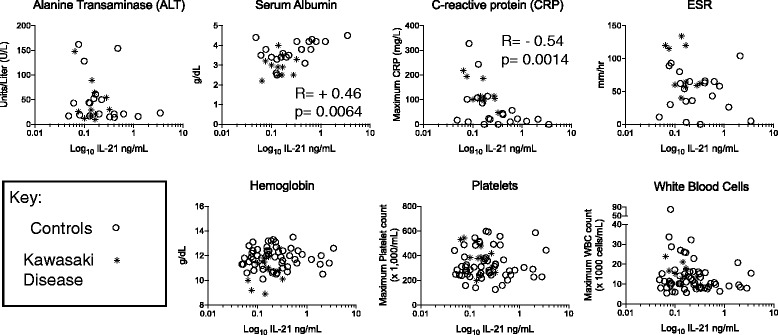
IL-21 level correlations with supplemental clinical indices used in diagnosing KD. IL-21 is graphed on a log^10^ scale to improve display of individual data points. Spearman *R* values are listed for the two correlations that reached significance: IL-21 correlations with lower CRP and higher albumin levels

To explore the possibility of the previous IL-21 elevation was related to a clinical marker rather than the KD state itself, we used Prism software (GraphPad, La Jolla, CA) to calculate Spearman R correlation coefficients between IL-21 levels and clinical indices (Fig. 3). Surprisingly, two markers used as supplemental criteria to support a diagnosis of KD showed significant correlations. However, the IL-21 levels were correlative in the opposite manner as one would predict if IL-21 was actually associated with KD. CRP is elevated in children with KD, but higher IL-21 levels are correlated with lower CRP levels in our patient cohort (Spearman R correlation value of 0.54 and two-tailed *p* value of 0.0014*). Lower albumin levels are also seen in KD, but higher albumin levels correlated with IL-21 elevation (Pearson *R* value of 0.46 with a two tailed *P* value of 0.0064*). ALT elevations can be seen in KD, and we similarly saw lower ALT levels associated with lower IL-21 levels, although this was underpowered and did not reach significance (Spearman *r* = −0.21; *p* = 0.26).

### IL-21 relation to systemic KD criteria

**Fig. 4:**
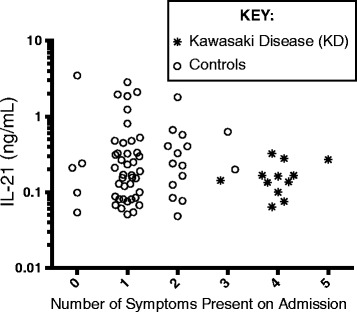
IL-21 levels relative to number of KD criteria symptoms. IL-21 levels (log^10^ scale to improve display of individual levels) are graphed relative to KD criteria: conjunctivitis, rash, mucous membrane changes, distal extremity changes, and solitary lymph node swelling. *Open circles* are controls and stars are KD samples

The telltale sign of KD is not solely the fever, but the systemic vasculitis that contributes to the number of findings that make up the criteria for diagnosis. These include conjunctivitis, polymorphous rash, mucous membrane inflammatory changes, distal extremity swelling, and solitary lymph node enlargement. Additional analysis was done to determine if the presenting clinical symptoms correlated with IL-21 levels. We analyzed the IL-21 levels by number of KD criteria present in both the KD children and controls (Fig. 4). Notably, only one of 12 KD children were incomplete and no controls had over three criteria either on admission notes or when evaluated by ID consultant. There was no significant difference in IL-21 levels between these groups, allowing us to conclude that specific symptoms do not correlate with IL-21 levels. The appearance of a trend in higher IL-21 levels with lower number of symptoms is driven by very few samples.

## Discussion

KD is a childhood vasculitis of unknown etiology, difficult to diagnose with certainty. Since recent studies implicate B cells in the pathogenesis of KD, and IL-21 is involved in the differentiation of both naïve and memory B cells into mature plasma cells [16], we were interested to see if IL-21 correlates with KD, as was previously shown [15]. Despite the small number of samples present in this study, the IL-21 levels in the KD group assayed with ELISA were similar to previously published reports. Our samples showed ranges of IL-21 levels in both KD and control samples that were consistent in repeated experiments, suggesting no inherent bias in the way our samples were processed. Our results do not support IL-21 elevation as a specific marker of KD in heterogeneous North American populations.

The findings published herein agree with limited data in a larger study on IP-10 in a Han Chinese cohort of children [17]. However, for the specific question of IL-21 levels, that study was hampered by a lack of sensitivity of the cytokine bead array (CBA) used. In our ELISA assay, all samples measured above our limit of detection, with a number in the thousands of pg/mL. Similarly, the data from the Korean cohort has data in the thousands of pg/mL with the majority of their samples in the measurable assay range of the ELISA. Of the 57 samples in the Han Chinese cohort assayed by CBA however, only six samples (two KD and four controls) had levels distinguishable from background levels with none of these levels being greater than 250 pg/mL. The lack of sensitivity of the CBA for IL-21 made conclusions from this study difficult to extrapolate. This lack of interpretable data necessitated our current study. We show a wide-range of IL-21 values in our samples and our median IL-21 level in the KD subgroup of 170 pg/mL was comparable to 499.5 pg/mL shown previously by ELISA in the Korean cohort of children [15].

IL-21 has a potential role in other inflammatory and autoimmune disorders, such as type 1 diabetes, allergy, and asthma [22], and inflammatory disorders such as the spondyloarthropathies [23]. Evidence supports IL-21 being involved in the transition from innate immune responses to specific T cell responses against antigens [13] so it would not be surprising for a number of infections to lead to IL-21 elevation as these results would support.

The prior study showing specific IL-21 elevation in KD in the Korean cohort was notable in that it included only suspected mononucleosis subjects in the control group. Since mononucleosis and KD both cause prolonged fever, we did evaluate if there was a bias in our study due to length of fever (Fig. 2). IL-21 in mononucleosis has not been well studied. The Epstein-Barr Virus, the most common cause of mononucleosis, does infect B cells directly [24] and some literature reports a delay in IL-21 production during Epstein-Barr Viral infection [20]. In light of our current results, it is reasonable to consider that the original study may be more significant for a lack of IL-21 elevation during evaluation for mononucleosis rather than for a specific elevation associated with KD.

Genetic background may partially explain the higher incidence and differences in clinical scoring systems in Asia compared to North America [4, 25]. These differences support that KD is more likely an immunological peri-infectious response, [26], rather than from direct invasion of the coronaries by an infectious agent [27]. The genetic differences may also partially explain the disparity of the results in the studies related to IL-21. Firm conclusions regarding genetic heterogeneity are hampered by the lack of a control group from a more homogenous population and lack of knowledge of the HLA status of subjects in this current study. These areas would be of interest in pursuing in future studies.

There are other significant limitations to this study. Firstly, and most obviously, we have few KD samples. However, the range of IL-21 levels between our KD group and the previously published Korean cohort are similar [15] and the main conclusion depends more significantly on the control group. The controls (60 subjects) shows a wide range of IL-21 elevations so we believe the conclusion that there is a non-specific IL-21 elevation is sound. Secondly, most of our control group would not have been clinically confused with KD as they lacked criteria beyond fever. A more ideal control group would be febrile children with four or five KD symptoms but given an alternative diagnosis. However, due to the number of KD cases also having co-infections with other organisms, a group such as this may be even more rare than children diagnosed with KD. Additionally, a number of the included controls would llkely have fulfilled criteria for incomplete KD (only 2 or 3 symptoms with supplemental laboratory support) without their alternative diagnosis. Thirdly, timing of inflammation may significantly affect cytokine levels as early inflammation may reflect the initial assault and later inflammation may be driven by repair. Ideally, serial samples starting early in the disease course would be obtained from subjects for these types of studies. Serial studies like this are difficult to obtain in children due to safety risk of multiple blood draws and parental concern over multiple needle sticks. Also, KD is not often recognized early in the course since the symptoms can be variable and confused with a myriad of viral and non-viral illness seen by general pediatricians. In one cohort of 152 children, only 20 were included on or before day three of illness [18]. We did attempt to address if timing of sampling affected the IL-21 levels (Fig. 2) and show no differences on that limited data set. Repetitive sampling over time, however, would better address this question.

## Conclusions

The marked predilection for acquired heart disease in children warrants research into determining the underlying physiology behind this vasculitis, both in terms of improved diagnostics as well as more appropriate treatment for typical, atypical, and incomplete KD. Unfortunately, particularly for the heterogenous populations of North America, IL-21 does not seem to be the answer. Future studies would benefit from larger cohorts, serial evaluation over time, and more extensive proteomic evaluation.

## Additional file

Additional file 1: Table S1. Laboratory values relative to clinical diagnosis classification. . (DOC 43 kb)

## Abbreviations

ALT: Alanine transaminase
AST: Aspartate aminotransferase
CRP: C-reactive protein
ESR: Erythrocyte sedimentation rate
GI: Gastrointestinal
IL-21: Interleukin-21
KD: Kawasaki Disease
IVIG: Intravenous immunoglobulin
PBMC: Peripheral blood mononuclear cell
WBC: White blood cell count

## References

[1] Matsuda H, Hashimoto N, Suzuki K, Aoyagi H, Akashi Y, Kawasaki K (2005). Long-term follow-up of a patient with Kawasaki disease and coronary aneurysm associated with asymptomatic thrombosis: a case report. J Cardiol.

[2] Burns JC, Shike H, Gordon JB, Malhotra A, Schoenwetter M, Kawasaki T (1996). Sequelae of Kawasaki disease in adolescents and young adults. J Am Coll Cardiol.

[3] Pinna GS, Kafetzis DA, Tselkas OI, Skevaki CL (2008). Kawasaki disease: an overview. Curr Opin Infect Dis.

[4] Newburger JW, Takahashi M, Gerber MA, Gewitz MH, Tani LY, Burns JC (2004). Diagnosis, treatment, and long-term management of Kawasaki disease: a statement for health professionals from the committee on rheumatic fever, endocarditis, and Kawasaki disease, council on cardiovascular disease in the young, American heart association. Pediatrics.

[5] Burns JC, Glode MP (2004). Kawasaki syndrome. Lancet.

[6] Newburger JW, Takahashi M, Gerber MA, Gewitz MH, Tani LY, Burns JC (2004). Diagnosis, treatment, and long-term management of Kawasaki disease: a statement for health professionals from the committee on rheumatic fever, endocarditis and Kawasaki disease, council on cardiovascular disease in the young, American heart association. Circulation.

[7] Yorifuji T, Tsukahara H, Doi H. Breastfeeding and Risk of Kawasaki Disease: A Nationwide Longitudinal Survey in Japan. Pediatrics. 2016. doi:10.1542/peds.2015-3919. 10.1542/peds.2015-3919 27244853

[8] Lee YC, Kuo HC, Chang JS, Chang LY, Huang LM, Chen MR (2012). Two new susceptibility loci for Kawasaki disease identified through genome-wide association analysis. Nat Genet.

[9] Chang CJ, Kuo HC, Chang JS, Lee JK, Tsai FJ, Khor CC (2013). Replication and meta-analysis of GWAS identified susceptibility loci in Kawasaki disease confirm the importance of B lymphoid tyrosine kinase (BLK) in disease susceptibility. PLoS One.

[10] Rowley AH, Shulman ST, Spike BT, Mask CA, Baker SC (2001). Oligoclonal IgA response in the vascular wall in acute Kawasaki disease. J Immunol.

[11] Rowley AH, Shulman ST, Garcia FL, Guzman-Cottrill JA, Miura M, Lee HL (2005). Cloning the arterial IgA antibody response during acute Kawasaki disease. J Immunol.

[12] Shingadia D, O’Gorman M, Rowley AH, Shulman ST (2001). Surface and cytoplasmic immunoglobulin expression in circulating B-lymphocytes in acute Kawasaki disease. Pediatr Res.

[13] Kasaian MT, Whitters MJ, Carter LL, Lowe LD, Jussif JM, Deng B (2002). IL-21 limits NK cell responses and promotes antigen-specific T cell activation: a mediator of the transition from innate to adaptive immunity. Immunity.

[14] Spolski R, Leonard WJ (2014). Interleukin-21: a double-edged sword with therapeutic potential. Nat Rev Drug Discov.

[15] Bae YJ, Kim MH, Lee HY, Uh Y, Namgoong MK, Cha BH (2012). Elevated serum levels of IL-21 in Kawasaki disease. Allergy, Asthma Immunol Res.

[16] Ettinger R, Sims GP, Fairhurst AM, Robbins R, da Silva YS, Spolski R (2005). IL-21 induces differentiation of human naive and memory B cells into antibody-secreting plasma cells. J Immunol.

[17] Ko TM, Kuo HC, Chang JS, Chen SP, Liu YM, Chen HW (2015). CXCL10/IP-10 is a biomarker and mediator for Kawasaki disease. Circ Res.

[18] Lee KY, Han JW, Hong JH, Lee HS, Lee JS, Whang KT (2004). Inflammatory processes in Kawasaki disease reach their peak at the sixth day of fever onset: laboratory profiles according to duration of fever. J Korean Med Sci.

[19] Lee KY, Rhim JW, Kang JH (2012). Kawasaki disease: laboratory findings and an immunopathogenesis on the premise of a “protein homeostasis system”. Yonsei Med J.

[20] Hagn M, Panikkar A, Smith C, Balfour HH, Khanna R, Voskoboinik I (2015). B cell-derived circulating granzyme B is a feature of acute infectious mononucleosis. Clin Transl Immunol.

[21] Vivanco-Cid H, Maldonado-Renteria MJ, Sanchez-Vargas LA, Izaguirre-Hernandez IY, Hernandez-Flores KG, Remes-Ruiz R (2014). Dynamics of interleukin-21 production during the clinical course of primary and secondary dengue virus infections. Immunol Lett.

[22] Leonard WJ, Spolski R (2005). Interleukin-21: a modulator of lymphoid proliferation, apoptosis and differentiation. Nat Rev Immunol.

[23] Andersen T, Rasmussen TK, Hvid M, Holm CK, Madsen KJ, Jurik AG (2012). Increased plasma levels of IL-21 and IL-23 in spondyloarthritis are not associated with clinical and MRI findings. Rheumatol Int.

[24] Kuppers R (2003). B cells under influence: transformation of B cells by Epstein-Barr virus. Nat Rev Immunol.

[25] Loomba RS, Raskin A, Gudausky TM, Kirkpatrick E. Role of the Egami Score in Predicting Intravenous Immunoglobulin Resistance in Kawasaki Disease Among Different Ethnicities. Am J Ther. 2015;23:e1293–9. 10.1097/MJT.0000000000000045 25611359

[26] Scuccimarri R (2012). Kawasaki disease. Pediatr Clin North Am.

[27] Rowley AH, Shulman ST (2010). Recent advances in the understanding and management of kawasaki disease. Curr Infect Dis Rep.

