# Supplementary material for: Observational study of Interleukin-21 (IL-21) does not distinguish Kawasaki disease from other causes of fever in children
Source: Pediatr Rheumatol Online J. 2017 Apr 20;15:32. doi: 10.1186/s12969-017-0163-3 (PMC5397673; doi:10.1186/s12969-017-0163-3)
Supplement: Additional file 1: Table S1. — Laboratory values relative to clinical diagnosis classification. . (DOC 43 kb) [file 12969_2017_163_MOESM1_ESM.doc]

**Additional file 1: Table S1 Laboratory values relative to clinical diagnosis classification**

| *Diagnoses:*  *(n= )* | | *Kawasaki Disease*  *(12)* | *All Controls^a^*  *(60)* | *Controls with CRP^b^ tested*  *(20)* | *Control Samples by Diagnosis* | | | | |
| --- | --- | --- | --- | --- | --- | --- | --- | --- | --- |
|  |  |  |  |  | *Bacterial Infections*  *(16)* | *Viral*  *Respiratory*  *(10)* | *Viral GI^c^*  *(8)* | *Viral Rash Prominent*  *(12)* | *Viral NOS*  *(10)* |
| *Laboratory Test* | *Units* | Median Laboratory Test Value  (number with available value within each group)  2.5^th^-97.5^th^ percentile | | | | | | | |
| White blood cells | x 10^6^/mL | 14.7  (12)  8.3-23.9 | 10.7  (56)  5.7-38.3 | 11.2  (20)  (5.7-32.2) | 16.2  (15)  6.4-32.2 | 10.4  (10)  7.9-79.4 | 10.9  (6)  6.2-20.8 | **9.4*****^d^*  **(12)**  **5.5-14.2** | **9.4****  **(10)**  **5.7-17.2** |
| Platelets | x 10^4^/mL | 386.0  (12)  197.0-544.0 | 297.0  (56)  138.6-593.5 | 365.0  (20)  140.0-598.0 | 310.0  (15)  140.0-598.0 | **246.5****  **(10)**  **126.0-488.0** | 266.5  (6)  226.0-442.0 | 319.5  (12)  159.0-557.0 | **272.0****  **(10)**  **216.0-372.0** |
| CRP | mg/L | 106.8  (11)  47.6-218.3 | **22.9****  **(20)**  **0.0-327.4** | **22.9****  **(20)**  **0.0-327.4** | 39.1  (5)  22.2-327.4 | 9.8  (1)  NA^e^ | 17.1  (1)  NA | **44.4****  **(7)**  **0.0-108.0** | 20.6  (3)  0.0-113.7 |
| Hemoglobin | g/dL | 11.0  (12)  8.9-12.6 | **11.9****  **(56)**  **10.5-13.3** | 11.6  (20)  10.5-13.1 | 11.7  (15)  10.1-13.2 | **12.2****  **(10)**  **11.3-13.5** | 11.6  (6)  11.3-12.3 | 11.6  (12)  10.5-13.1 | **12.0****  **(10)**  **10.6-12.8** |
| ESR^f^ | mm/hr | 62.0  (11)  0.0-134.0 | 56.0  (20)  0.0-104.0 | 58.0  (19)  0.0-104.0 | 62.0  (5)  35.0-93.0 | 43.0  (1)  NA | 11.0  (1)  NA | 63.0  (7)  0.0-89.0 | 36.0  (3)  3.0-65.0 |
| Hematocrit | % | 32.1  (12)  26.7-36.5 | **34.8****  **(56)**  **30.4-39.9** | **34.1****  **(20)**  **31.3-38.4** | 37.3  (15)  31.6-39.5 | **35.2****  **(10)**  **33.0-41.0** | 34.5  (6)  32.2-37.4 | 34.5  (12)  30.4-38.4 | **35.4****  **(10)**  **30.3-37.2** |
| AST^g^ | U/L | 27.0  (11)  14.0-109.0 | **48.0****  **(19)**  **23.0-128.0** | **47.0****  **(14)**  **23.0-96.0** | 43.0  (4)  30.0-68.0 | 32.0  (1)  NA | 46.0  (1)  NA | 48.0  (7)  23.0-83.0 | 79.0  (4)  25.0-128.0 |
| ALT^h^ | U/L | 29.0  (11)  10.0-148.0 | 23.0  (19)  14.0-162.0 | 22.0  (14)  14.0-162.0 | 30.0  (4)  14.0-43.0 | 19.0  (1)  NA | 17.0  (1)  NA | 21.0  (7)  15.0-162.0 | 55.5  (4)  21.0-128.0 |
| Albumin | g/L | 2.9  (12)  0.0-4.0 | **3.8****  **(21)**  **2.5-4.5** | **3.8****  **(16)**  **2.5-4.5** | 3.5  (5)  2.5-4.2 | **4.3****  **(2)**  **4.2-4.3** | 4.4  (1)  NA | **3.8****  **(7)**  **2.6-4.2** | **3.5****  **(4)**  **3.4-3.6** |

^a^ ”All Controls” includes two non-febrile individuals and two inflammatory/rheumatic patients; ^b^ C-reactive protein- CRP; ^c^ GI- gastrointestinal; ^d^ **Significant difference from KD (p < 0.05) are in **bold font;** ^e^ NA- not available; ^f^ erythrocyte sedimentation rate- ESR; ^g^ aspartate aminotransferase- AST; ^h^ alanine aminotransferase- ALT.
